# Are in vitro and in silico approaches used appropriately for animal-based major depressive disorder research?

**DOI:** 10.1371/journal.pone.0233954

**Published:** 2020-06-24

**Authors:** Constança Carvalho, Susana A. M. Varela, Tiago A. Marques, Andrew Knight, Luís Vicente

**Affiliations:** 1 Faculdade de Ciências, Centro de Filosofia das Ciências, Universidade de Lisboa, Lisboa, Portugal; 2 Faculdade de Ciências, cE3c – Centre for Ecology, Evolution and Environmental Changes, Universidade de Lisboa, Lisboa, Portugal; 3 Centre for Research into Ecological and Environmental Modelling, University of St Andrews, St Andrews, Scotland; 4 Departamento de Biologia Animal, Faculdade de Ciências, Centro de Estatística e Aplicações, Universidade de Lisboa, Lisboa, Portugal; 5 Centre for Animal Welfare, University of Winchester, Winchester, United Kingdom; National Center for Toxicological Research, UNITED STATES

## Abstract

The current paradigm for biomedical research and drug testing postulates that *in vitro* and *in silico* data inform animal studies that will subsequently inform human studies. Recent evidence points out that animal studies have made a poor contribution to current knowledge of Major Depressive Disorder, whereas the contribution of *in vitro* and *in silico* studies to animal studies- within this research area- is yet to be properly quantified. This quantification is important since biomedical research and drug discovery and development includes two steps of knowledge transferability and we need to evaluate the effectiveness of both in order to properly implement 3R principles (Replacement, Reduction and Refinement). Here, we used the citation tracking facility within Web of Science to locate citations of original research papers on *in vitro* and *in silico* related to MDD published identified in PubMed by relevant search terms. 67 publications describing target papers were located. Both *in vitro* and *in silico* papers are more cited by human medical papers than by animal papers. The results suggest that, at least concerning MDD research, the current two steps of knowledge transferability are not being followed, indicating a poor compliance with the 3R principles.

## 1. Introduction

Biomedical research heavily relies on animal studies, despite the ethical and clinical limitations of these [1].

**Fig 1:**
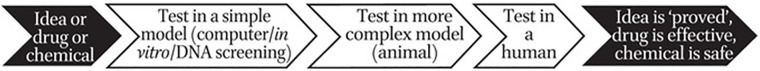
Current paradigm of biomedical research and drug discovery and development. Kindly provided by Taylor [2].

The standard contemporary paradigm for biomedical research, and drug discovery and development, requires scientists to test putative new clinical interventions, by progressing from simple to increasingly complex models, prior to conducting human studies and trials, as shown in Fig 1.

Even though this paradigm is more focused toward drug discovery, it is also encouraged for broader research, by legislation and guidelines pertaining to animal research, in various countries and regions (e.g.[3]).

Supporters of animal studies within biomedical research claim that 1) it is not possible to discontinue their use, as that would jeopardize human health, and that 2) human-based methods (*in silico* and *in vitro*) are used in early steps of biomedical research to inform the animal research community, hence avoiding unnecessary or excessive use of animals. For example, purportedly, if a substance shows high levels of toxicity *in vitro* it will not progress into animal testing [4]. In the same way, a drug that shows high toxicity in animal testing should not proceed to human trials. However, it has been demonstrated that human trials may sometimes occur simultaneously with animal trials, rather than sequentially, as one would expect if animal trials were an essential step prior to human trials [5].

In our previous study we compared the number of citations *in vitro*, *in silico* and non-human primate-based (NHP) original studies focused on Major Depressive Disorder (MDD), that were received (i) in total, (ii) by unspecified human medical papers, and (iii) by human medical papers focused on MDD. We verified that both *in vitro* and *in silico* research papers received more citations by human medical papers, than NHP papers. This was unexpected, considering that most countries restrict the use of NHPs, making it reasonable to presume that when they were used, they should provide a significant contribution to human health. However, this was not the case. Data obtained via simpler models (*in vitro* and *in silico*) seemed to be more visible or considered more important by the human medical research community. This called into question the contemporary paradigm of biomedical research and drug discovery, in which knowledge is presumed to transfer between animal and human models [6].

Considering that this paradigm presumes two steps of knowledge transferability: i) between simpler and complex models, and ii) between animals models and humans, we wondered if there could be knowledge transferability problems in step (i), similar to those we demonstrated at step (ii).

Hence, the aim of the current study is to assess whether *in vitro* and *in silico* papers describing original data on a human disorder (MDD) are being appropriately cited by subsequent animal-based papers. It is important to mention that animal models are extensively used in MDD research. In fact, by the time our study was conducted there were about twice as many original papers using animal models in MDD research than papers using *in vitro* and *in silico* approaches.

During studies focused on MDD, animals frequently undergo severe procedures such as learned helplessness or forced swim test protocols. Most applicable legislations and guidelines mandate that such procedures should be avoided wherever possible. Hence it is reasonable to expect that the MDD-focused animal research community should be particularly alert to the data and insights provided by simpler data.

Even though there is a wide consensus that the use of simpler models such as *in vitro* and *in silico* methods within basic and applied biomedical research helps animal researchers to meet the principles of Replacement (of animals with alternatives) and Reduction (of animal numbers), as described by Russell & Burch [7], to our knowledge, there has never been a systematic study that empirically verifies whether animal researchers are, indeed, applying this principles to their practice *i*.*e*. if they are locating and using applicable data obtained via such simpler models.

If *in vitro* and *in silico* studies are indeed seen as an important step prior to conducting animal studies in biomedical research, and animal studies are in turn seen as important prior to conducting human studies, then we would expect that papers describing *in vitro* or *in silico* data on a human disorder should be cited more frequently by animal papers, than by human medical papers. If, on the contrary, this is not the case, then further studies on other human disorders and drug development should be conducted to confirm the extent to which the contemporary theoretical paradigm for biomedical research is actually being followed in practice. If adherence is not as common as believed, then this paradigm should clearly be revised.

## 2. Methods

We conducted a citation analysis as defined by Garfield and Merton [8]. Concisely, in a citation analysis, target papers are located first and then a search for all other papers citing the former is performed.

The information compiled comprises the total number of citations, and the patterns of citation. We used a total of 67 target papers of *in vitro* or *in silico* studies on MDD- utilising only human data, selected from the citation analysis database created in our previous study [6]. The citation analysis was performed between September 2016 and June 2017. We considered all published papers using *in vitro* or *in silico* methods, that aimed to gain knowledge about MDD, and were published prior to 2011, to enable five-year time for citations–a frequently used timeline for citation analysis [9]. To locate target papers we searched PubMed–the largest freely accessible bibliographic database, using the following Medical Subject Heading (MeSH) search terms: ‘Depressive Disorder, Major’ AND (“*in silico*” OR ‘computer model’ OR ‘mathematical model’ OR ‘computer simulation’ OR ‘*in vitro’* OR ‘cell culture’ OR ‘culture technique’ OR ‘cell line’ OR ‘organ culture’ OR ‘tissue culture’. Our goal was to select original publications that presented new data, so we used PubMed filters to exclude review articles (“review”, “systematic review”, “meta-analysis”, “bibliography”) as well as opinion articles (“biography”, “autobiography”, “comment”, “editorial”, “interview”). We also excluded by hand *in vitro* papers that used animal tissue or cells. Using the citation tracking facility within Web of Science, we counted the number of times each target paper was cited by subsequent papers in the following categories: `animal research papers`, `human medical papers`, `*in vitro* papers`, and `*in silico* papers`. Citing papers may have been assigned to more than one category if they described different research approaches (e.g. human-based and *in vitro*).

## 3. Results

In total, 464 (18%) of the 2,574 citations received by the 38 *in vitro* papers were by invasive animal research papers, and 978 (40%) were by human medical papers. For the 29 *in silico* papers, 44 (5%) of the 806 citations were by invasive animal research papers, and 317 (39%) by human medical papers.

**Fig 2:**
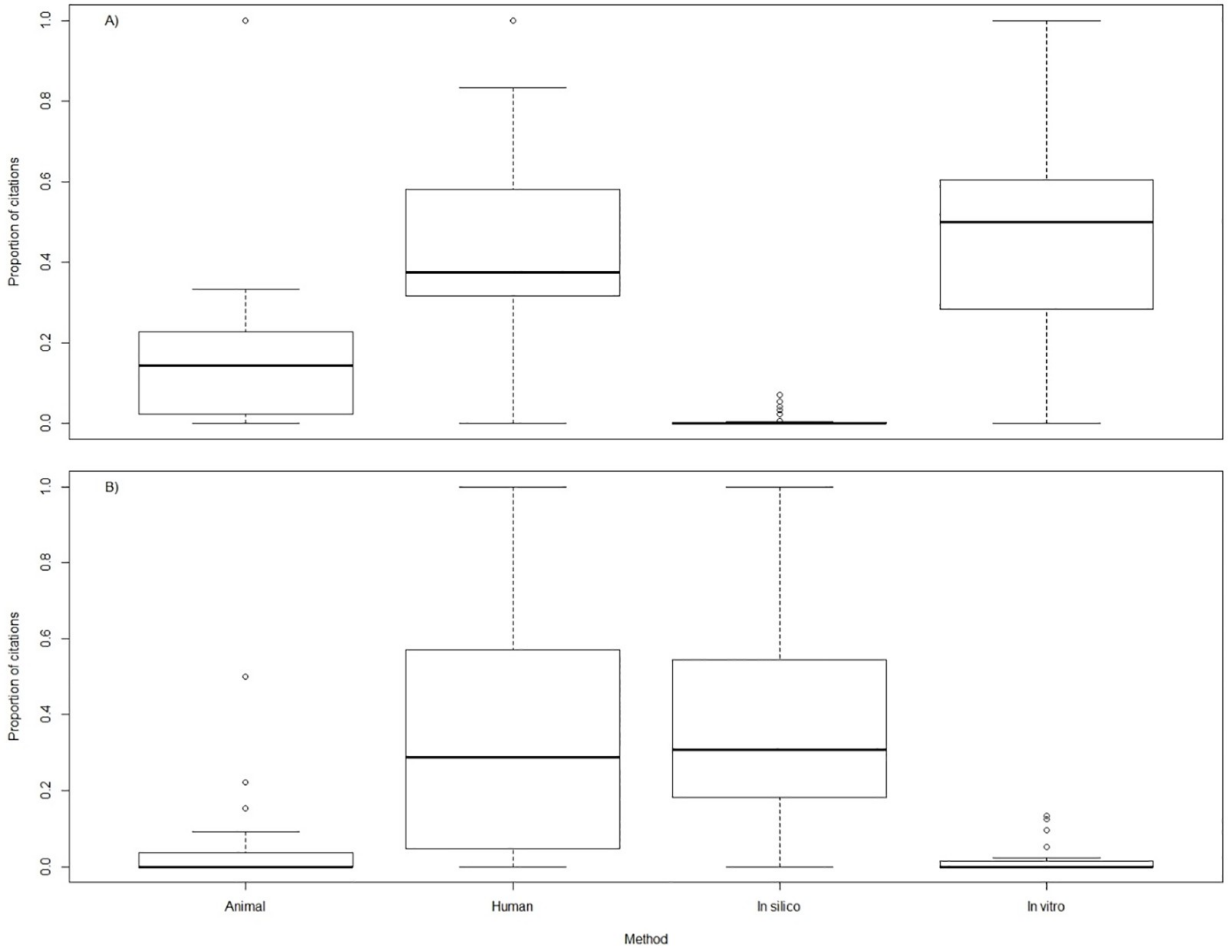
Boxplots of the proportion of citations received by research category for *in vitro* A) and *in silico* B) papers on MDD.

As shown in Fig 2, the majority of citations received by both *in vitro* or *in silico* target papers were by papers employing the same research method, and by human medical papers. The proportion of citations by animal papers and the other research method were considerably lower. More importantly, the proportion of citations by animal papers was lower than by human medical papers.

## 4. Discussion

The results of our citation analysis suggest that the standard approach to testing medical hypotheses–which postulates that *in vitro* and *in silico* research is an important step prior to conducting animal testing–is not supported by citation data, at least for MDD research. Clearly, MDD biomedical research utilising *in vitro* and *in silico* data does not seem to be considered important by, or at least more important to, the animal research community, than it is to the human medical community.

One can argue that if the animal research community is not citing *in vitro* and *in silico* papers on MDD, these might be of limited use. However, that is inconsistent with their substantial use by the human medical community, which cites more this kind of research than research based on animal studies [6]. Additionally, this lack of transferability of knowledge between the animal and the human medical research communities is further evidenced by the fact that, in general, most citations received by animal research papers are within other animal-based studies, rather than within human medical papers [10].

MDD is a complex human mental disorder with multifactorial aetiopathogenesis [11], so one cannot extrapolate that the citation patterns found here will necessarily be replicated in other disorders that have just one cause (e.g. Down’s syndrome). Furthermore, a single disease analysis is not enough to generalize the results to the entire field of biomedical research.

Hence, the next step should be the use of a similar approach targeting monofactorial disorders and drug trials. If, as whole, these studies produce similar results, then it would be compelling evidence that the accepted paradigm for biomedical research and drug discovery and development is not being sufficiently followed, which supports the claims made by several authors [1] that the 3Rs are not being addressed as well as required by applicable legislation and good research practice. This suggests that animal studies in biomedical research are mostly defining their research priorities autonomously, rather than being perfectly framed in the biomedical research paradigm.

Sixty years ago Russell and Burch [7] established the foundations of much current legislation regarding animal experimentation, with the formulation of the 3R principles. Even though the research community unanimously welcomes them, the focus of their application has predominantly been refinement, and not always in an effective way [1].

Nowadays there is an increasing number of databases on human and animal protein expression differences (for a review see [12]) which, on the one hand, makes it easier for researchers to locate and cite existing data; but, on the other hand, might stimulate animal research to be conducted independently of *in vitro* and in *silico data* to populate such databases.

In theory, the reduction principle depends upon the standard use of *in silico* and *in vitro* techniques prior to animal studies. If original data on human disorders from *in vitro* and *in silico* approaches are not being used by the animal research community, then the reduction principle is not being properly fulfilled. The reasons behind this must surely be multiple.

One of the possible reasons is the inadequacy of systematic reviews that animal researchers sometimes perform on their research topic, prior to conducting animal experiments. These should prevent unnecessary animal use [13], but by excluding from the search *in vitro* and *in silico* studies, researchers can exclude an important source of knowledge.

Based on our results we recommend that changes are made in current systematic review protocols in order to include *in vitro* and *in silico* data.

Another reason that became salient with our study and deserves attention, is that *in vitro* and *in silico* approaches are, by definition, human-based methods, not animal-based methods. Conceivably human data is not relevant enough for animal papers, in the same way animal studies do not seem to be relevant to subsequent human studies [6,10].

This highlights that the current paradigm of biomedical research and drug discovery and development includes two steps of knowledge transferability between the animal and the human models, neither of which appear to work well. If similar results are found in other disorders and more importantly, in drug discovery, than the current paradigm must be changed. Specifically, animal testing must be deprioritized, with greater investment in human-based *in vitro* and *in silico* research approaches.

## Supporting information

S1 File (ODS)

## References

[1] Herrmann K. Refinement on the way towards replacement: Are we doing what we can? In Herrman K, Jane K, editors. Animal Experimentation: Working Towards a Paradigm Change. Boston: Brill; 2019 pp. 1–64. 10.1163/9789004391192

[2] Taylor K. Recent developments in alternatives to animal testing In Herrman K, Jane K, editors, Animal Experimentation: Working Towards a Paradigm Change. Boston: Brill; 2019 pp. 583–609. 10.1163/9789004391192_002

[3] Workman P, Aboagye EO, Balkwill F, Balmain A, Bruder G, Chaplin DJ, et al Guidelines for the welfare and use of animals in cancer research. British Journal of Cancer. 2010; 102(11): 1555–1577. 10.1038/sj.bjc.6605642 20502460 PMC2883160

[4] Choudhuri S, Patton GW, Chanderbhan RF, Mattia A, Klaassen CD. From classical toxicology to Tox21: Some critical conceptual and technological advances in the molecular understanding of the toxic response beginning from the last quarter of the 20th century. Toxicological Sciences. 2017;161(1): 5–22. 10.1093/toxsci/kfx186 28973688 PMC5837539

[5] Pound P, Ebrahim S, Sandercock S, Bracken MB, Roberts I. Where is the evidence that animal research benefits humans? BMJ. 2004; 328(7438): 514–517. 10.1136/bmj.328.7438.514 14988196 PMC351856

[6] Carvalho C, Varela SAM, Bastos LF, Órfão I, Beja V, Sapage M, et al The Relevance of *In silico*, *In vitro* and Non-human Primate based Approaches to Clinical Research on Major Depressive Disorder. ATLA. 2019a; 47 (3–4): 128–139. 10.1177/0261192919885578 31838868

[7] Russell WMS, Burch RL. The principles of humane experimental technique. London: Methuen; 1959.

[8] Garfield E, Merton RK. Citation indexing: Its theory and application in science, technology, and humanities (Vol. 8). New York: Wiley; 1979.

[9] Wooding S, Pollitt A, Castle-Clarke S, Cochrane G, Diepeveen S, et al Mental Health Retrosight: Understanding the returns from research (lessons from schizophrenia): policy report. Rand health quarterly 2014 4(1). Available et https://www.rand.org/pubs/research_reports/RR325.html. 10.7249/rhq-04-01-08 PMC5051976 28083322

[10] Carvalho C, Alves D, Knight A, Vicente L. Is animal-based biomedical research being used in its original context? In: Herrman K, Jane K, editors. Animal Experimentation: Working Towards a Paradigm Change. Boston: Brill; 2019b pp. 376–390. 10.1163/9789004391192_017

[11] Chiriţă AL, Gheorman V, Bondari D, Rogoveanu I. Current understanding of the neurobiology of major depressive disorder. Rom J Morphol Embryol. 2015;. 56(2 Suppl): 651–658. 26429155

[12] Yin JY, Sun W, Li FC, Hong JJ, Li XX, et al VARIDT 1.0: Variability of Drug Transporter Database. Nucleic Acids Research. 2020; 48(D1): D1042–D1050. 10.1093/nar/gkz779 31495872 PMC6943059

[13] Leenaars M, Hooijmans CR, van Veggel N, Ter Riet G, Leeflang M, Hooft L, et al A step-by-step guide to systematically identify all relevant animal studies. Laboratory Animals. 2012; 46(1): 24–31. 10.1258/la.2011.011087 22037056 PMC3265183

